# Detection of visual field defects using Eye Movement Pediatric Perimetry in children with intracranial lesions: feasibility and applicability

**DOI:** 10.1016/j.heliyon.2022.e11746

**Published:** 2022-11-17

**Authors:** Najiya Sundus K. Meethal, Jasper Robben, Deepmala Mazumdar, S. Loudon, N. Naus, J.R. Polling, J. van der Steen, Ronnie George, Johan J.M. Pel

**Affiliations:** a Department of Neuroscience, Vestibular and Ocular Motor Research Group, Erasmus MC, Rotterdam, The Netherlands; b Medical Research Foundation, Chennai, India; c Department of Ophthalmology, Erasmus MC, Rotterdam, The Netherlands; d Royal Dutch Visio, Huizen, The Netherlands

**Keywords:** Eye movement perimetry, Pediatric perimetry, Saccadic reaction time, Goldmann visual field, Intracranial lesions

## Abstract

The study aimed at evaluating the feasibility of Eye Movement Pediatric Perimetry (EMPP) among children in detecting Visual Field Defects (VFDs) associated with Intracranial Lesions (IL). Healthy controls (n = 35) and patients diagnosed with IL (n = 19) underwent a comprehensive clinical evaluation followed by a Goldmann Visual Field (GVF) and a customised EMPP protocol. During EMPP, all the participants were encouraged to fixate on a central target and initiate Saccadic Eye Movement (SEM) responses towards randomly appearing peripheral stimuli. The SEM responses were recorded using an eye-tracking device and further inspected to calculate Performance Scores (PS), Saccadic Reaction Times (SRTs), and an EMPP Index (EMPI). The mean age (years) of the controls and cases were 7.3 (SD: 1.5) and 9.4 (SD: 2.4) respectively. Among the controls, the older children (≥7 years) showed statistically significantly faster SRTs (p = 0.008) compared to the younger group. The binocular EMPP measurements compared between the controls and the cases revealed no statistically significant differences in PS (p = 0.34) and SRT (p = 0.51). EMPP failed in 4 children because of data loss or unacceptably poor PS whereas GVF failed in 7 children due to unreliable subjective responses. Of the 16 reports, with regard to the central 30-degree VF, 63% of the outputs obtained from both methods were comparable. EMPP is a reliable method to estimate and characterise the central 30-degree VF in greater detail in children with IL. EMPP can supplement the conventional methods, especially in those children who fail to complete a long duration GVF test.

## 1 Introduction

Children diagnosed with Intracranial Lesions (IL) need a multidisciplinary approach to treatment and monitoring. These children commonly present with Visual Field Defects (VFDs) particularly when the lesions involve the visual pathway and the nature of the lesion dictates its location and extent. Hence perimetry turns out to be an integral component in the ophthalmological management protocol to evaluate the stability or progression of the causative lesion. Perimetry not only contributes to clinical management decisions but also offers essential assistance while rehabilitating these children [1, 2].

Regardless of the development of Standard Automated Perimetry (SAP) for adults, there are still limited possibilities for perimetry in children. Goldmann Visual Field (GVF) technique is a recommended kinetic approach in children that helps to identify the alterations in the Visual Field (VF) shape/area and delineate quadrant/hemifield defects [3, 4, 5]. In GVF, the VF is manually mapped by instructing the participant to remain fixated on the central cue located in the middle of a hemispherical bowl. Meanwhile, a circular light stimulus is gradually moved from the extreme of the projection surface into the field in order to mark the boundaries and establish the isopter. GVF is very efficient in evaluating the periphery beyond 30°, relying on the multiple possible responses from the patient along the entire trajectory and the sequence of kinetic scanning. This aids in identifying sharp-edged scotomas or steep isopter boundaries as seen in classic VFDs associated with neuro-ophthalmic conditions [3]. But the drawback is that there is no consensus or standard protocol to conduct the procedure. Therefore, GVF demands a highly-skilled examiner who can accurately manipulate the stimulus presentation and interact well with the participants for gaining consistent subjective responses. Thus the procedure is subjected to substantial outcome variabilities induced by the examiner as well as by the patient. The requirement of prolonged fixation and attention makes it challenging to obtain reliable and repeatable test results in children [3, 4, 5]. Moreover, small sensitivity changes and widespread or diffuse VFDs are difficult to identify using this method. Regardless of its limitations, GVF is still recommended as it permits the evaluation of a wide extent of VF and delivers valuable clinical information in children with neuro-ophthalmic disorders.

In view of the above-mentioned challenges, we evaluated Eye Movement Perimetry (EMP) as an alternative approach of automated perimetry in children with neurological impairment. Previous studies have shown the applicability of EMP among adults in detecting glaucomatous VFD [6, 7, 8] as well as in the assessment of visual orienting behavior in young children [9, 10], and in infants [11]. EMP relies on natural human reflexes where the detection of static peripheral stimuli is indicated by making prosaccades. From each goal-directed Saccadic Eye Movement (SEM), the system calculates the Saccadic Reaction Time (SRT). A combination of both these outcomes results in a reliable clinical index for VF mapping. Here, we marginally modified the EMP setup used for adult patients [6, 7] into an EMPP method (Eye Movement Pediatric Perimetry).

The aim of the current study was to investigate the applicability of EMPP in VF estimation among children with IL for which we included a subset of children diagnosed with IL with and without the genetic mutation of Neurofibromatosis-type 1 (NF-1). The EMPP incorporates a customised VF test protocol integrated with Eye Tracking Technology (ETT) to capture real-time fixation and SEM responses to VF stimuli. The outcome measures from the EMPP testing were the pattern of SEM responses (seen/unseen/invalid), a score calculated from the number of reliable responses, named as Performance Score (PS), and the SRT calculated from ‘reliably seen responses. This study addressed the following objectives: (a) to evaluate the feasibility of the EMPP in children, (b) to compare the PS and SRT between a control group and a set of children diagnosed with IL (c) to describe the applicability of EMPP in predicting the functional deficits associated with the lesions in comparison with the GVF outputs.

## 2 Materials and methods

### 2.1 Study participants

To evaluate the feasibility and practicability of the EMPP method in the pediatric population, a set of healthy pediatric participants (n = 35) aged between four to ten years were recruited from a regular primary school in Rotterdam, The Netherlands. This healthy control group was included when the following criteria were met (1) born between 37 to 42 weeks of gestation, (2) no significant history of visual or ocular problems, and (3) no evidence of brain injury/damage.

To explore the clinical value of EMPP, a patient group who was diagnosed with definite IL (with and without genetic mutation of NF-1) with or without clinically evident functional defects on GVF perimetry was included. Patients were recruited from the outpatient clinic of the pediatric ophthalmology department at the Erasmus MC, Rotterdam, The Netherlands. Since the reliable and successful completion of a perimetry test necessitates visual attention and concentration, the patient group specifically included children diagnosed with the genetic mutation of NF-1. Apart from the wide-ranging physical complications, NF-1 is also characterised to cause diverse cognitive dysfunctions including reduced intelligence scores, compromised visuospatial abilities, and attention deficits that might make the perimetry challenging or influence the results [12, 13, 14]. Hence the enrollment of this subgroup aided us to explore if the EMPP approach can be successfully applied to children with neurodevelopmental disabilities. Due to the outbreak of COVID-19, only 19 patients were requested to participate in the present study. Parents or legal guardians provided written informed consent before the commencement of the study to access the children's medical registers and to include the video recording of the experimental session. The essential information related to the visual status such as reduced Visual Acuity (VA) and presence of VFDs recorded using GVF perimetry (such as hemianopia or quadrantanopia), evidence of behavioral comorbidities such as Attention Deficit Disorders (ADD), Autism Spectrum Disorder (ASD), developmental delay, and Intellectual disability were extracted from the medical records. Experimental procedures were approved by the medical ethical committee of the Erasmus University Medical Centre, Rotterdam, The Netherlands (MEC-2017-1150), and adhered to the declaration of Helsinki [15].

### 2.2 Goldmann Visual Field (GVF) perimetry

GVF perimetry was conducted by a skilled perimetrist who manually mapped the VF by projecting circular light stimuli of six stimulus sizes (ranging from 0.0625 mm^2^ to 64 mm^2^). The stimulus was moved from the non-seeing area into the seeing area (field of vision) and whenever the patient saw the stimulus, the response buzzer was pressed and the perimetrist marked the corresponding point. At the conclusion of the procedure, the marks were connected by a smooth line to form the isopter. GVF reports were obtained for each of the patients along with the related details such as Pupil Diameter (PD) in mm (manually measured using a diameter gauge), refractive correction, quality of the subjective responses (good/slow/varying), and reliability of the central fixation (good/moderate/bad).

### 2.3 EMPP: measurement setup and procedures

The EMP setup and the test paradigm that was developed and evaluated in the adult population were transformed marginally for making it suitable for the pediatric population [6, 7]. The modifications were as follows: (a) the chin rest installed in the EMP used for the adult population was removed while examining the children thereby giving them an unrestricted and naturalistic environment. This was feasible as the Tobii tracker compensates for head motion within some boundaries, (b) the adult EMP test protocol projected white circular visual stimuli (Goldmann size III) at four different stimulus intensity levels, whereas for the children smiley faces at a single intensity level were used to encourage attention and retain interest, and (c) a webcam recording was added to monitor the child's overall performances, attentiveness, and eye movement behaviour.

**Figure 1:**
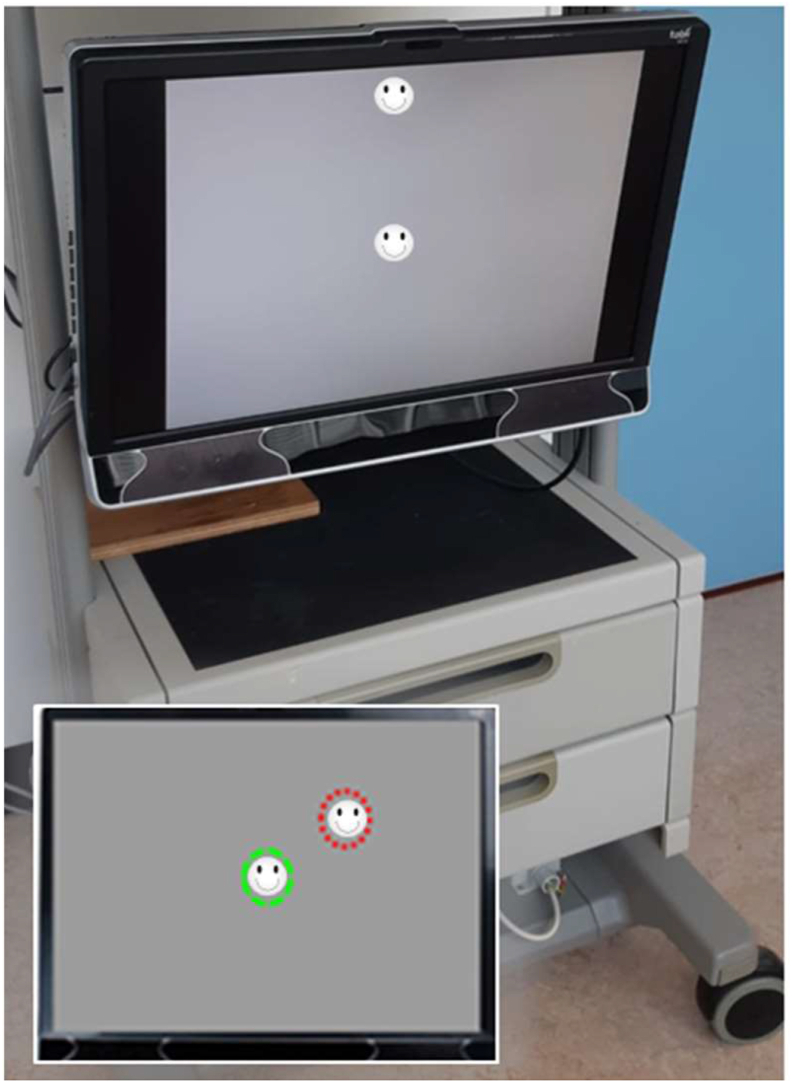
EMPP set up with a 24-inch display monitor integrated with an infrared eye-tracking device and a height/viewing angle adjustable surface housing. The inset picture shows an illustration of the smiley fixation target (circled with green dotted lines) and a peripheral smiley stimulus (circled with red dotted lines).

**Figure 2:**
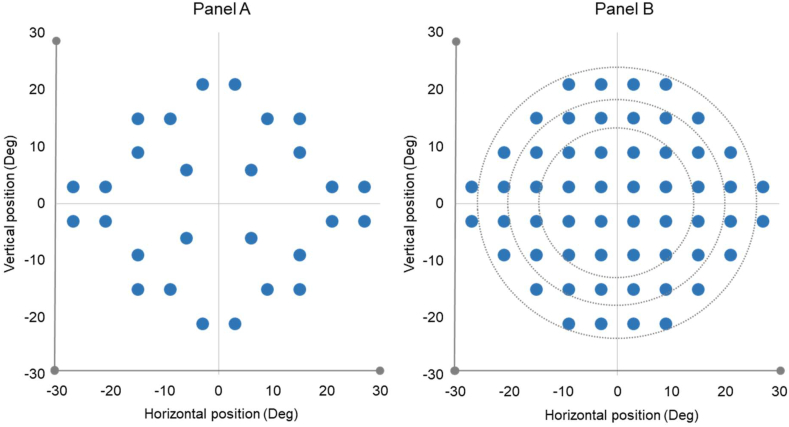
(A) Illustration of the screening grid with 28 test locations and of the full-field grid with 56 test locations. (B) Along with the zonal divisions based on stimulus eccentricity. x and y axis denotes the horizontal and vertical coordinates of the VF test locations in degrees (deg).

The EMPP measurement for the control and patient group differed in certain aspects. For the controls, the EMPP setup comprised of a 24-inch display monitor (test distance ∼60 cm) combined with an external and portable remote eye tracking bar (Tobii X3-120; Tobii, Sweden). Patients (Figure 1) were tested using a 24-inch monitor with an integrated infrared 60 Hz eye-tracking system (Tobii T60 XL; Tobii, Sweden). For the controls, the customised binocular VF test protocol relied upon a test grid that consisted of 28 locations (Figure 2: Panel A, adapted from Meethal NK et al., 2018). The total test duration was ∼2 min [6] and a maximum of 28 eye movement responses were obtained per individual. Meanwhile, in the patients, a more refined grid was used to detect the VFD that consisted of 56 locations (Figure 2: Panel B, adapted from Mazumdar et al., 2019). Each patient was tested under three different conditions: binocularly (OU) and monocularly (OD and OS) with adequate breaks in between. This approach took ∼4 min per measurement [7] and a maximum of 168 eye movement responses were recorded per individual. Every measurement series began with a binocular measurement so that the children familiarised themselves with the testing procedure followed by the monocular measurements, the order of which was randomised. Both the control group and the patient group performed EMPP measurements in a quiet and dim-lit room with no external interruptions. The parents or guardians were instructed to remain quiet during the measurement. In spite of the measurements being rapid (∼4 min), adequate breaks (minimum of 5 min) were given in between the measurements to eliminate the component of fatigability.

The EMPP test protocol was initiated with a preliminary tracking status estimation to ensure appropriate eye alignment and uninterrupted gaze tracking. This was followed by a standardised five-point calibration procedure to obtain optimal gaze accuracy. This step was repeated either for specified locations or for all five locations if a poor calibration was noted due to blink artifacts, poor focusing, or any hardware/software related glitches. Only after a successful calibration, the EMPP test was commenced by projecting a central fixation target (Figure 1) and peripheral visual stimuli (at a fixed intensity level) were projected sequentially at random test locations based on an overlap paradigm. The participants were instructed to fixate the central stimulus and look at any detectable stimuli in the periphery and refixate the central stimulus, while the eye tracker was simultaneously recording the eye movements. The natural reflexive eye movement initiated towards a detected peripheral stimulus was considered as a positive response instead of a verbal response or a response buzzer. Participants were instructed not to search for the peripheral stimuli. The peripheral stimuli were projected for a maximum duration of 1.2 s (s) on the screen. A random interval of 1–2 s was set between stimuli to prevent the predictability of stimulus appearance. For each of the presented stimuli, gaze data was collected and stored for post-analysis.

### 2.4 Eye movement data analysis and creation of VF plots

**Figure 3:**
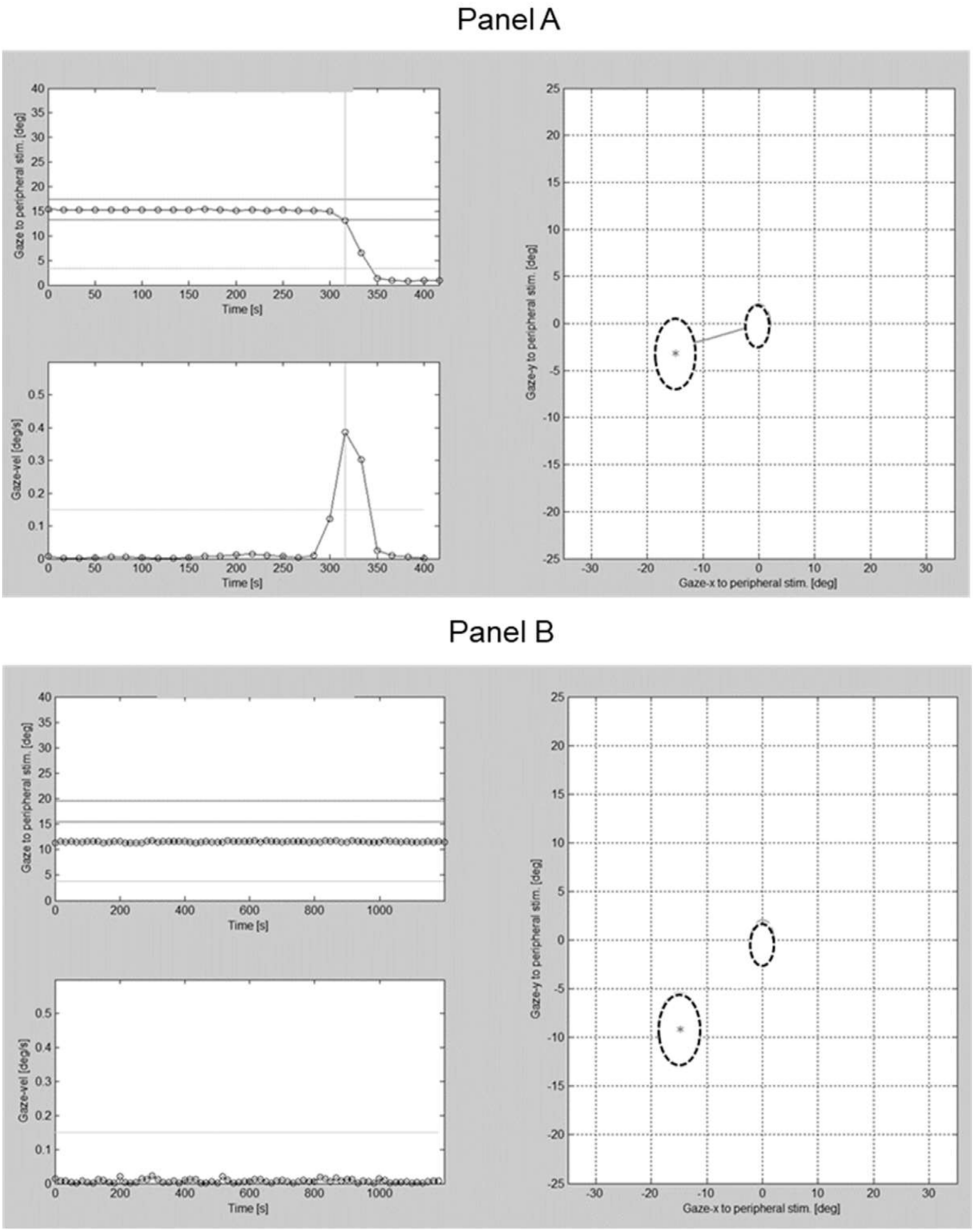
(A) Illustration of the Matlab window displaying an eye movement initiated from the central fixation to a peripheral stimulus (Right) and the gaze position map used to estimate the Saccadic Reaction Time (Top eft), and the corresponding gaze velocity (Bottom left). (B) Illustration of the Matlab window displaying no eye movement initiated from the central fixation to a peripheral stimulus (Right) and the corresponding gaze position map (Left).

The trajectory and time course of an SEM initiated towards a peripheral stimulus were analysed using a customised Matlab program (Mathworks Inc., Natick, MA, USA). For each trial, the SEM performance was assessed on the basis of various parameters and was visually inspected and labeled as ‘seen’, ‘unseen’, or ‘invalid’ (Figure 3). For each peripheral stimulus, a circular area of interest was defined with a radius of 6° and if the gaze trace crossed the border of this area the stimulus was identified as ‘seen’. To ensure the reliability of the ‘seen’ response the following criteria were used: (a) a steady fixation of the central stimulus was followed by a primary SEM directed towards the peripheral stimulus, (b) the angular difference between the direction of the primary SEM and the peripheral stimulus location was ≤45°, and (c) the amplitude of the primary SEM covered more than half (50%) of the total target distance. Whenever these criteria were not followed, a peripheral stimulus was classified as ‘unseen’. An event where inadequate eye movement data was obtained due to blinking or pupil tracking failure was labeled as ‘invalid’ and any trial with ‘invalid’ responses >25% was excluded from the final analysis.

A measure of the performance called PS (%) was calculated (ranging from 0 to 100% - least reliable to most reliable) from the number of reliable responses divided by the overall tested locations. The outcome measure for VF responsiveness, the SRT, was calculated from each of the ‘seen’ responses as the time between the onset of a peripheral stimulus and the initiation of the SEM which was done based on the gaze velocity criterion (the eye velocity ≥50°/s). Furthermore, a clinically utilisable index seemed necessary that can be grossly compared with the Visual Field Index (VFI) displayed in the standard VF reports of the Humphrey Field Analyser (HFA). Here, we introduced an EMP Index (EMPI) as an aggregate percentage of visual function for a given VF including the test locations where a visual response is estimated [16]. EMPI was calculated from responses with reliable SRT divided by the total reliable responses (excluding invalids). As in the VFI, the central VF locations were given relatively higher weightage compared to the mid-perioral and peripheral VF areas for which a weightage factor was introduced depending upon the eccentricity (degrees) of the stimulus locations (Figure 2: Panel B). The weightage factors were 0.5, 0.3, and 0.2 for central, mid-peripheral, and peripheral zones respectively, hence ranging the EMPI on a percentage scale of 0–100% (perimetrically blind to normal).

To demonstrate the integrity of the VF we generated two customised EMP plots per patient. The first plot, the PS plot, displayed the eye movement response at each tested location as ‘seen’, ‘unseen’, or ‘invalid’ (small empty circles). ‘Seen’ and ‘unseen’ test locations were denoted using filled-in circles with RGB shades of Grey (217-217-217) and black (0-0-0) respectively. The second plot named the Saccadic Reaction Time (SRT) plot displayed the SRT values corresponding to the ‘seen’ locations. SRT values from 200 to 1200 ms were illustrated using an RGB scale ranging from 230 to 25. The maximum duration window provided for a participant to respond reliably to a projected stimulus was 1.2 s (1200 ms), hence the maximum SRT on the scale was 1200 ms.

### 2.5 Statistical analysis

In order to address the first research question that focused on evaluating the feasibility of the EMPP method in the pediatric population, EMPP performance (seen, unseen, and invalid) and SRT values of the control group were described and compared by subdividing them into two age groups i.e. < 7 and ≥7 years (Independent t-test). In addition, for each age group, the effect of stimulus eccentricity on SRTs was tested for significance by using one-way ANOVA. To explore the applicability of EMPP in predicting the functional deficits concomitant with IL, EMPI and SRT values were compared between the patients (Non-NF-1 and NF-1) and the age-matched controls (Independent t-test). Since the test grid used for both the groups was different, this comparison was done by considering only those common test locations (n = 24) that are shared between the 28-point (controls) and 56-point (cases) test grids. A subgroup of healthy controls was assessed for age-matched comparison with the patient group. In the patient group EMPI and SRT values were descriptively compared between monocular (OD and OS) and binocular viewing (OU) conditions for both Non NF-1 and NF-1. To evaluate the clinical usefulness of EMPP a two-by-two contingency table was used to compare the gross agreement of ‘normal’ and ‘abnormal’ VF status shown by the GVF and the EMPP plots.

## 3 Results

A total of 54 pediatric participants were recruited that included 35 healthy controls (n = 20 females) and 19 cases (n = 8 females) with IL. In 2 controls (6%) and in 2 cases (11%), the EMPP measurement failed because of eye tracking data loss due to blinking or poor eye-tracking quality. The mean age of the resulting 33 controls was 7.3 (SD: 1.5) years and that of the 17 cases was 9.4 (SD: 2.4) years. The latter group consisted of lesions associated with (n = 8) and without (n = 9) the genetic mutation of NF-1.

### 3.1 Feasibility of EMPP in the control group

**Figure 4:**
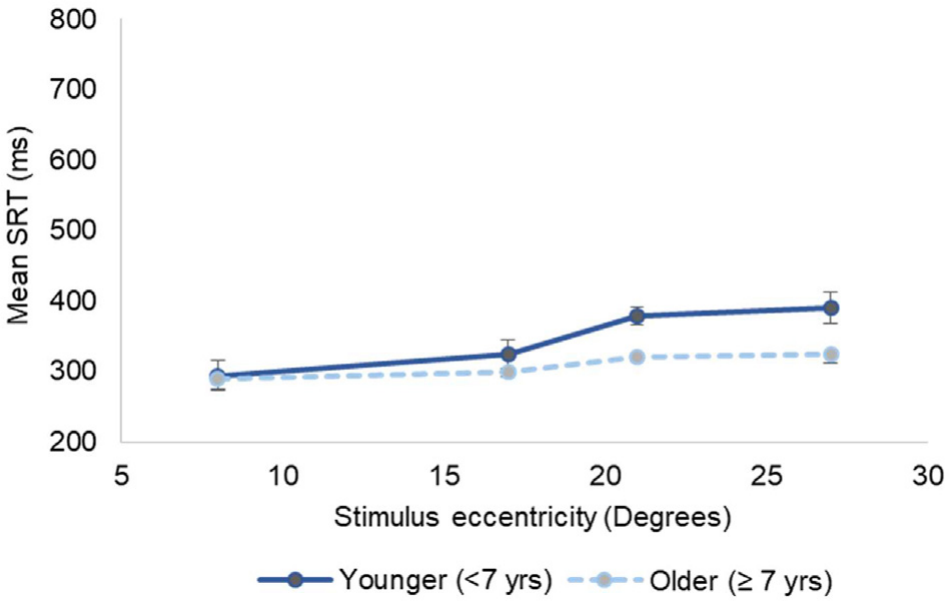
The Saccadic Reaction Time (SRT) plotted against stimulus eccentricity for younger and older children illustrating the delay in SRT with increasing eccentricity. The error bars represent Standard Error (SE).

In the control group, 3 children had an unacceptably low EMPP Performance (PS ≤ 10%), hence they were excluded from the analysis. A total of 717 reliable SEM responses were obtained from the control group that was used to calculate the mean SRTs. The older children (≥7 years) showed an equal PS and statistically significantly faster SRT values (p = 0.008, Independent t-test) when compared to the younger group (Table 1). Next, in both the groups of children (older children and younger), stimulus eccentricity had a significant effect on SRT (One-way ANOVA) with p-values < 0.001 and 0.03 respectively (Figure 4).

**Table 1:** Binocular PS and SRT value comparison between the older and younger children in the healthy control group.

Healthy controls (n = 30)	<7 years (n = 11)	≥7 years (n = 19)	p-value∗
Age range (years)	4–6	7–10	NA
Mean age (SD) in years	6.0 (0.9)	8.1 (1.1)	
Male: Female	3:8	7:12	
No: of reliable SEM responses obtained (n)	240	477	
Mean PS (SD) in %	98 (3)	98 (2)	1.00
Mean SRT (SD) in ms	344 (106)	311 (91)	0.008

∗ Independent t-test.

### 3.2 EMPP applicability in the patient group

The binocular EMP measurements (Independent t-test) compared between the age-matched controls (n = 17) and the cases (n = 17) on the basis of 24 common tested locations revealed no statistically significant difference in PS (p = 0.67) and SRT (p = 0.51) between the groups. The patient group comprised children diagnosed with IL associated with Non NF-1 and NF-1 mutations. There was no statistically significant difference between the mean age of both subgroups (p = 0.67). Table 2 describes the demographics and the clinical diagnosis of the Non NF-1 sub group and the type of neuro-developmental disabilities detected in the NF-1 group.

**Table 2:** Description of the cases/patient category including the Non NF-1 and NF-1 subgroups and their clinical diagnosis. ∗ADHD – Attention Deficit Hyperactivity Disorder, ASD – Autistic Spectrum Disorder.

Patient category (n = 17)	Gender ratio (male: female)	Mean age (SD) in years	Clinical diagnosis
Non NF-1 (n = 9)	3:6	9.3 (2.7)	1. Craniopharyngioma (n = 4) 2. Pilocytic astrocytoma (n = 3) 3. Chiasmal neuritis (n = 1) 4. Low-grade thalamic glioma (n = 1)
NF-1 (n = 8)	6:2	9.1 (2.1)	1. Neurofibroma (n = 4) 2. Optic nerve Glioma (n = 3) 3. Astrocytoma (n = 1)
			Neuro- developmental disabilities
			1. ADHD∗ (n = 1) 2. ASD∗ (n = 1) 3. ASD suspect (n = 1) 4. Developmental delay (n = 2) 5. Attention deficits (n = 1) 6. Mild intellectual disability (n = 2)

**Figure 5:**
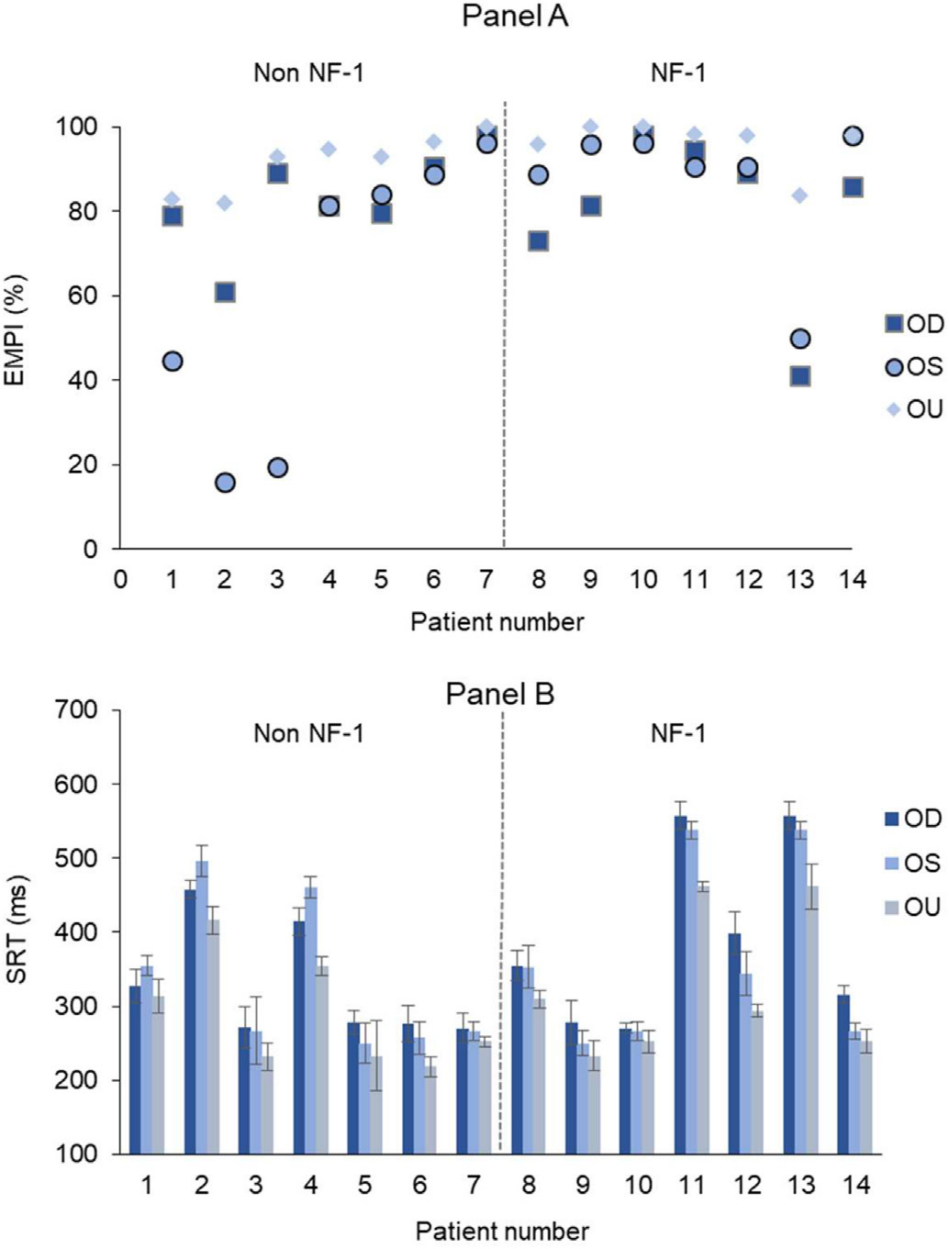
(A) Scatter plot illustrating the improvement in Eye Movement Perimetry Index (EMPI) and (B) fastening of Saccadic Reaction Time (SRT) values during monocular and binocular viewing conditions among the patient group. Patient number 1 to 7 belongs to the non NF-1 and 2 to 14 to NF-1 subgroup and the dotted line separates the subgroups for a visual comparison.

Three of the 17 patients who exhibited unacceptably poor performance on EMP (PS ≤ 10%) during either monocular or binocular testing conditions were excluded from the analysis which compared the performance between the two subgroups (Non NF-1 and NF-1) under monocular and binocular conditions. In total 1738 and 1859 reliable SEM responses obtained from the Non NF-1 and the NF-1 groups respectively were used to calculate the EMPI and the mean SRT values. At a sub group level, the mean monocular and binocular EMPI (Figure 5: Panel A) and SRT (Figure 5: Panel B) were comparable between the Non NF-1 (patient number 1 to 7) and NF-1 subgroups (patient number 8 to 14). Overall, the patient group showed a relatively improved EMPI ranging from 0% to 60% (Figure 5: Panel A) and faster SRTs (Figure 5: Panel B) in binocular viewing conditions when compared to monocular responses.

### 3.3 Evaluation of the clinical usefulness of EMPP

**Figure 6:**
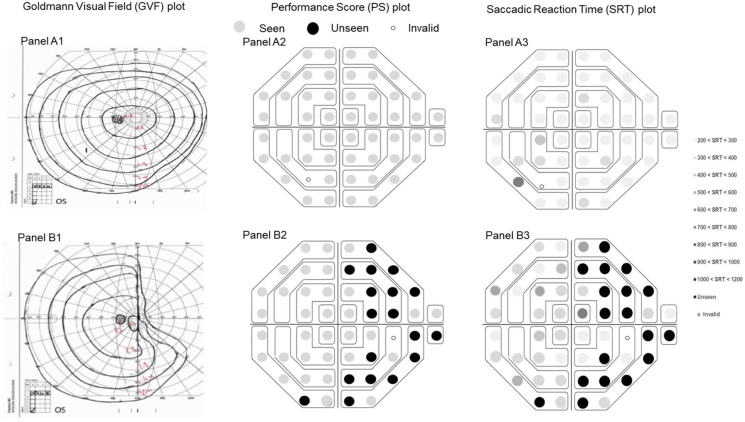
(A1 to A3) A comparison of GVF (Panel A1) and PS and SRT EMPP plots (Panel A2 and A3) illustrating a normal visual field on GVF as well as on the EMP. (Panel B1 to B3) A comparison of GVF (Panel B1) and PS plot and SRT EMPP plots (Panel B2 and B3) illustrating an abnormal visual field with the presence of nasal field loss with a residual temporal field. The scaling used for the PS and SRT plot are provided.

Overall 8 children were capable to reliably perform both the EMPP and GVF procedures and among those 16 reports, a moderate agreement was found between the two perimetry methods (kappa: 0.52, p = 0.02). 11 outputs were agreeing with each other on the basis of the presence and extent of VFD (An example is displayed in Figure 6).

## 4 Discussion

This study evaluated the feasibility of EMPP in healthy children and its clinical applicability in children diagnosed with IL. The customised method could be successfully performed by 94% of the controls and 89% of the cases, which included even children under four years of age. The testing method seemed sound among children as they were not required to maintain a constant fixation but instead were encouraged to perform a natural reflexive reaction in response to randomly appearing visual stimuli. In contrast to the EMP test setup implemented among the adults, EMPP was modified in such a way that the children were not required to place their heads in a fixed position because the data provided by the eye tracking system was compensated for limited head rotation. This gave a similar ambiance as that of electronic gaming with which most children were familiar. Our cases included children with NF-1 gene mutation who presented with diverse cognitive/developmental dysfunctions including ADHD, ASD, and intellectual disability. Since the preparation of eye movement requires processes including the shift of visual attention to the new stimuli and disengagement of oculomotor fixation we evaluated the subgroup with NF-1 mutation in specific [12, 13, 14]. Irrespective of the neuro-developmental disabilities, we found the NF-1 group to complete EMPP measurements with a promising and equal success rate in comparison with the Non NF-1 subgroup. The test failure that occurred in two controls and two cases was due to poor eye tracking quality and the measurements were not considered for the final analysis.

This study included a control group to essentially evaluate the feasibility and practicability of the proposed EMPP method in the pediatric population. Since these control children were enrolled in a regular primary school, we needed a portable eye-tracking test setup with a rapid and accurate VF evaluation protocol (binocular screening grid with 28 test locations). As a next step, cases were recruited for comprehensively evaluating the clinical applicability, and here we conducted monocular and binocular testing protocols (full-field with 56 test locations). The usage of a full-field grid (comparable to that of the HFA 24–2 protocol) made it possible to obtain extensive and relevant clinical information on the central VF. The screening grid locations were a subset selected from the full-field grid and the testing environment, decision algorithm, and analysis strategies were kept uniform to ensure comparability between the groups. In spite of the increased number of test points, we didn't anticipate a fatigability component to influence the EMPP performance as the test was rapid with a maximum test duration of ∼4 min. Still, the adequate breaks (minimum of 5 min) provided between the measurements ensured the elimination of the factor of fatigability. Moreover, the PS (%) reflected the level of EMPP performance and the calculated values didn't show any effect of fatigability in both the test cohorts.

Among the controls, the older children (≥7 years) showed comparable PS (%) but significantly faster SRT values when compared to that of the younger group. SRTs also showed a dependency on the stimulus eccentricity which was more pronounced in the younger group of children. This response pattern was on par with the literature that described the nature of saccade development throughout childhood [17, 18] and also the dependency of SRT on stimulus eccentricity though it was evaluated in a group of adults [19]. Among the cases, the improved eye movement responses during binocular measurement when compared to the monocular condition were similar to the eye movement behaviour observed among adults with glaucoma (unpublished data from the current study group) and also with contrast sensitivity thresholds on Humphrey Field Analyser [20]. SRT values were statistically comparable between the controls and cases probably due to the inclusion of only binocular responses on the basis of common tested locations.

This study showed that EMPP can detect the presence and extent of expected VFD associated with the brain lesions with a moderate inter-method agreement in comparison with GVF. A previous study with a comparable methodology to the current study evaluated the clinical applicability of a novel technique called Saccadic Vector Optokinetic Perimetry (SVOP) in detecting VFD associated with brain tumors in children [10]. Similar to EMPP, the SVOP system also integrates eye-tracking technology with a customised test paradigm. But instead of their gap paradigm, the current study used an overlap paradigm as SRT is reported to be influenced by the fixation task [21]. Similar to SVOP, EMPP is also a viable method for VF testing in children to detect the presence and extent of VFD, with a possibility for monocular as well as binocular measurements. In addition to a binary response plot (seen/unseen), EMPP has an SRT plot for displaying the VF responsiveness, thereby offering supplemental clinical information. In EMPP, each test location was tested once. In our previous papers, we reported the benefits of an interactive protocol, which we applied to patients with glaucoma [6, 7]. This approach not only makes the test faster but also allows for an ongoing check for SEM responses from the fixation target to the peripheral visual stimuli. Here in EMPP, each test location was tested once, but the customisable protocol offers the possibility to repeat testing/crosschecks for pre-selected test locations. Hence in the future, we recommend further refinement of the protocol by incorporating this interactive approach between the participant and the test paradigm.

The stimulus grid used in EMPP is extended to a total visual angle of 54° horizontally and 42° vertically, hence could characterise the central VF area in greater detail (≤30 deg VF). GVF and EMPP outputs showed only a moderate agreement, and the maximum proportion of disagreement (25%) occurred due to the presence of VFD detected in the SRT plot of EMPP (generalised/localised delays in SRT) whereas GVF showed a relatively normal VF. This mandates a further comparison of EMPP outputs with the VF outcome predicted in correspondence to the structural abnormalities detected using Optical Coherence Tomography and Magnetic Resonance Imaging. A single report which presented with a peripheral field loss beyond the central 30° was categorised as normal by EMPP whereas GVF identified a functional loss. Similarly, only 64% of the cases had a decline in central VA in combination with poor EMPI, hence it is evident that alternative approaches to VF testing are not a replacement for the current clinical standards. Instead, it is an additional source of supplementary information. A calculation of EMPI for the central test locations (within 10°) would be worthwhile to compare this functional index with central VA. A follow-up study would be beneficial to evaluate the scope of using eye movement based parameters as prognostic indices among patients diagnosed with IL.

## 5 Conclusions

EMPP is a feasible method for estimating the VF among children with IL. The empirical plots of EMPP could detect and characterise the central 30-degree VF in greater detail thereby complementing GVF perimetry. In the future, a formal evaluation of its diagnostic ability in comparison with structural defects detected using imaging modalities might be beneficial.

## Declarations

### Author contribution statement

Najiya Sundus K. Meethal, Jasper Robben: Performed the experiments; Analyzed and interpreted the data; Wrote the paper.

Deepmala Mazumdar: Performed the experiments; Analyzed and interpreted the data.

S. Loudon, N. Naus, J. R. Polling, J. van der Steen: Conceived and designed the experiments; Contributed reagents, materials, analysis tools or data.

Ronnie George: Analyzed and interpreted the data; Contributed reagents, materials, analysis tools or data.

Johan J. M. Pel: Conceived and designed the experiments; Analyzed and interpreted the data; Contributed reagents, materials, analysis tools or data.

### Funding statement

This work was supported by Glaucoomfonds, Oogfonds, Rotterdamse Stichting Blindenbelangen, and Stichting Blindenhulp contributed through UitZicht (Grant number: Uitzich 2017–23).

### Data availability statement

Data will be made available on request.

### Declaration of interest's statement

The authors declare no conflict of interest.

### Additional information

No additional information is available for this paper.
